# How Will COVID-19 Impact on the Governance of Global Health in the 2030 Agenda Framework? The Opinion of Experts

**DOI:** 10.3390/healthcare8040356

**Published:** 2020-09-23

**Authors:** Luis A. Fernández-Portillo, Antonio Sianes, Francisco Santos-Carrillo

**Affiliations:** 1 Department of Business Management, Universidad Loyola Andalucía, 14004 Córdoba, Spain; portillo@uloyola.es; 2 Research Institute on Policies for Social Transformation, Universidad Loyola Andalucía, 14004 Córdoba, Spain; 3 Department of International Studies, Universidad Loyola Andalucía, 14004 Córdoba, Spain; frsantos@uloyola.es

**Keywords:** Global Health, public health systems, 2030 agenda, sustainable development goals, COVID-19, institutional design, content analysis, NVIVO

## Abstract

In 2015, the 2030 Agenda was formally adopted by the United Nations, establishing a set of 17 Sustainable Development Goals (SDGs). SDG 3 seeks to promote Global Health and the quality of public health systems in developing countries. The achievement of these goals requires the commitment of all signing countries, but the COVID-19 crisis is changing the behavior of the main stakeholders in the international arena. What do the experts think about these changes? Could these changes threaten the 2030 Agenda and Global Health? To answer these questions, we conduct a content analysis of 152 documents written by experts from the 15 main think tanks on international development policy. The results point out that the new scenario brought about by the pandemic is hindering the necessary cooperation between countries to achieve global health goals and to guarantee public health coverage in developing countries. To deal with these challenges, more delegation of powers to international organizations and a reform of the international cooperation system are needed. With this analysis, we launch a warning about potential weaknesses of the institutional design of the 2030 Agenda in order for it to survive in a post-COVID-19 world and remain a valid instrument to promote health worldwide.

## 1. Introduction

In 2015, the United Nations General Assembly formally adopted the 2030 Agenda for Sustainable Development, establishing a set of 17 Sustainable Development Goals (SDGs) to be pursued until 2030 [1]. The 2030 Agenda has been considered a remarkable achievement of the international community, as it was able to arrive at an important consensus on the need to move towards a more inclusive and sustainable development model [2].

The Agenda incorporates Global Health as one of its main objectives, (SDG 3), to “Guarantee healthy lives and promote well-being for all at all ages” [1]. However, by virtue of its transversal nature [3], Global Health is mentioned throughout the entire Agenda under the guiding principle that “no one should be left behind”.

Even though the SDGs are expressed as a collective action [4,5], they must be implemented at the national level, on a nonbinding basis, in accordance with the objectives and plans of action established by each state [6,7]. For this purpose, the 2030 Agenda merely provides a set of general guidelines for its final design and implementation. Nevertheless, it is assumed that states will keep to the commitments adopted regarding multilateral governance of the Agenda, and that both these commitments and governance are not going to change. The COVID-19 crisis has undermined both these assumptions.

The enormous uncertainty that the recent COVID-19 crisis has thrust upon the current international order means that we are now facing a more unclear global scenario. So far, the response to the crisis has been mainly promoted at the national level, while a lack of international coordination has been the rule [8,9]. Even within the European Union (EU) there has been a growing reluctance to cooperate [10]. From a rational point of view, therefore, there are more than obvious reasons to believe that the pillars of international cooperation and governance that sustain the 2030 Agenda, in general, and specifically Global Health, are seriously threatened in a world that appears much more vulnerable.

In terms of the SDG on Global Health, progress towards the objectives will require a strong effort by individual states to include key aspects of the 2030 Agenda, such as universal health coverage in their territories. However, it will also involve a solid commitment to the multilateral agreements reached by the international community, and a more effective governance to maintain cooperation [2,11]. Many studies have already analyzed the relevance of the 2030 Agenda for the achievement of Global Health at planetary level. On the one hand, the promotion of multilateral governance on Global Health issues [12], the role given by the Agenda to civil society and other private actors [13] or the framework it offers to guide action [14] have already been deemed as crucial for the achievement of Global Health. On the other hand, the international cooperation system introduced by the 2030 Agenda is necessary for developing countries to achieve their own national objectives, be it in terms on maternal health care [15] or health equity [16,17].

COVID-19 has not yet disappeared, and some voices are already warning about a more virulent new outbreak of the virus that will strain even more, if possible, the health systems of most countries, but especially the more vulnerable. Nevertheless, whether it be COVID-19 or other diseases, international society has begun to accept the idea that the world we live in has changed, and such international agreements as the 2030 Agenda are at risk. Experts, politicians and other actors are currently identifying how and where that change is taking place and how it will affect global balance and the positioning of individual states in the international arena.

With these ideas in mind, our study pursues two objectives; first, to shed some light on how the COVID-19 pandemic could affect the behavior of individual actors and threaten their commitments to international cooperation, jeopardizing the implementation of the 2030 Agenda and the achievement of Global Health for all; second, to analyze how the institutional design of the 2030 Agenda could be affected by the COVID-19 crisis and how it should be adapted in consequence, according to the experts’ view.

## 2. Materials and Method

### 2.1. Research Design

In this paper, we deal with the opinion of experts about the impact that the COVID-19 pandemic will have on the governance of Global Health in the 2030 Agenda framework. The research questions that will guide this analysis include the following. What is the opinion of experts on international development regarding the new global situation? What do they think about the reactions of governments and the impact of the changes introduced due to the pandemic on the international order? According to their view, could these changes impact the promotion of Global Health, in particular, and other SDGs in this uncertain future?

To answer these questions, we conducted a content analysis of more than 150 documents written by experts from the 15 main think tanks on International Development Policy, according to the Global Go to Think Tank Index Report [18]. It is crucial to understand these points of view, as the influence of epistemic communities and experts on political decisions has been widely addressed by the academic literature. Today, there is strong evidence that supports how policies have been informed or influenced in their design by the opinion emerging from such experts, e.g., in the United States [19], the United Kingdom [20], China [21], Brazil [22] and at global level [23].

To gather the expert opinion needed to perform such analysis, we rely on the methodological procedure explained in the following subsection. To systematize opinion, we follow the categories introduced by the functionalist rational approach to institutional design.

### 2.2. Sources and Data Collection

#### 2.2.1. Sources

To conduct expert-based research, one of the first topics to address is that of the selection criteria to identify such experts. In recent times, think tanks have been widely used because of their direct influence on political processes. The role of think tanks is to provide policymakers with analysis and expert opinion, based on facts, figures and rigorous research. They were established with the aim of persuading policymakers to take their ideas on board and eventually to shape their agenda [24]. The privileged position occupied by think tanks in public debate is reflected in their access to mainstream media and their political commentary, frequently accepted as independent expert opinion [25]. In fact, in recent years, think tanks do not simply observe or advise on policy, but have also become involved in policy delivery [19,26].

Every year, the Think Tanks and Civil Societies Program at the University of Pennsylvania rates think tanks worldwide, and its findings are presented in the Global Go to Think Tanks Index Report [18]. The report rates think tanks in different categories, such as Defense and National Security, Education Policy, and Social Policy, among others. To gather the voice of the experts regarding the expected impact of COVID-19 on the state of the world and the future achievement of the Global Health goal of the 2030 Agenda, we focus on think tanks under the International Development Policy category, as ranked in the 2019 Edition. The top 15 think tanks, plus the Center of Excellence, for the period 2016–2018, from which we gather our database, are introduced in Table 1.

#### 2.2.2. Period

The period of analysis focuses on the vivid early debate on the consequences of COVID-19 for the state of the world, mainly generated once the coronavirus had hit the Western world. Even though there are pertinent contributions before this period, and they have continued afterwards, the database includes documents from 12 March–16 April 2020. The starting date was chosen as the day after the World Health Organization declared COVID-19 to be a global pandemic, which influenced the declaration and diffusion of a state of alarm/exception in most European countries, gathering attention from many experts in international development policy. Regarding the closing date, we wanted to highlight short-term reactions to the pandemic, as the relevance of our analysis relies on early reactions to the unexpected shock to the international equilibrium. Thus, we intended to include only declarations and statements for a one-month period. However, the relatively later arrival of the COVID-19 debate in the United States (US) and in most developing countries suggested a short extension, so the search covers contributions made until 16 April.

#### 2.2.3. Language

Only contributions written in English were included. English is the dominant language in the international debate and the language used by most think tanks on international development. Internal reasons also existed for this decision, as a clear systematization that could allow for comparisons was necessary. Documents originally written in another language but translated into English by think tanks were also considered to diversify the range of opinions. 

#### 2.2.4. Final Database

**Figure 1:**
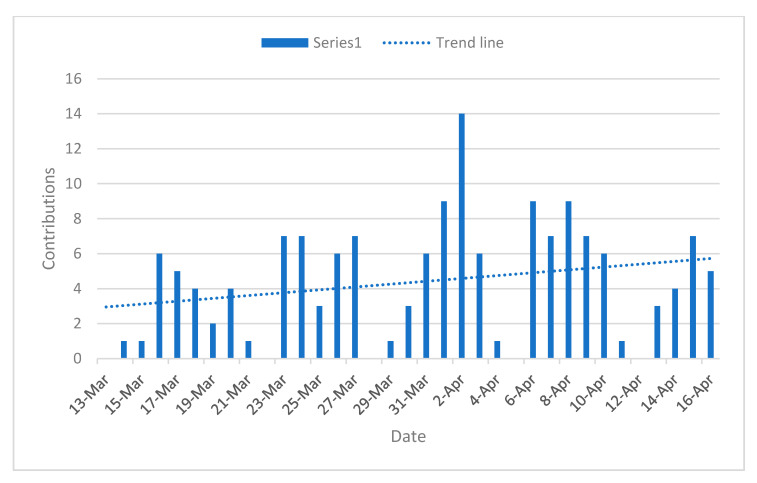
Contributions per day from 14 March to 16 April.

Once the search strategy was performed, we gathered a total of 152 documents according to the selection criteria. After the analysis, 119 documents provided information relating to our categories that could be used for the analysis. The remainder were used to enrich our understanding of the context. For the rest of the paper, we will refer to the documents by their code, which will appear in brackets from (1) to (152). Information about these documents and their authors can be found as Table A1 in the Appendix A. Figure 1 shows the contributions made per day. As think tanks barely publish during weekends, there is a periodical pattern of publication. However, as the trend line reveals, contributions have followed a slight linear growth.

### 2.3. Methodology and Analytical Approach

#### 2.3.1. Argument Mining and NVIVO as a Tool for Systematization

Content analysis has been defined as a research technique that objectively, systematically and quantitatively describes the content of communication [27] (p. 18). This technique is widely used to analyze different politics-related topics [28,29], specifically health-policy issues [30]. One of the available techniques to systematize the opinions of the group being studied via content analysis is argument mining. Argument mining is a field of corpus-based discourse analysis that has the goal of automatically identifying argumentation structures in a discourse [31].

With this goal, the identification of types of argument is crucial, which requires the use of software assisted by human coders to identify co-occurrences of individual arguments. In our study, we perform content analysis using NVIVO 12 Plus (QSR International, Melbourne, Australia), software commonly employed in qualitative research [32,33,34].

A compulsory step in analyzing the documents is the identification of categories (or “codes”, in NVIVO terminology). Each relevant text passage is linked to one category and codified as such. This procedure allows the retrieval of all passages codified under each category and the performance of different types of query, such as those included in our study (see Table 2 and Table 3 in the results section). It is important to note that the same passage may be codified under different categories. 

To arrive at an organized picture of the major changes that the COVID-19 crisis is bringing about in the context of Global Health in the 2030 Agenda framework, it is necessary to identify the main dimensions in which these changes are occurring and the relationships between them. In our paper, we have opted for the functional rationalist approach to the design of international agreements such as the Agenda. This framework was developed by Koremenos et al. [35] and Koremenos [36], and the reasons that have encouraged this choice are addressed in the following subsection.

#### 2.3.2. The Rational Approach to the Design of International Agreements as a Framework

By institutional design, we understand the definition of norms, rules, procedures, and organizational structures that enable and constrain behavior and action so as to achieve desired objectives or to execute given tasks [37]. The institutional design approach applies to international agreements such as the 2030 Agenda, embedded in the study of global governance. This field has lately experienced growing prominence due to the significant increase in institutionalized cooperation of a transnational nature [38,39].

Due to the importance of analyzing the institutional design of international agreements, the field is not free of debate. For rationalist authors, the institutional design of international agreements is the rational response to exogenous problems, as the result of deliberate interactions between states and other international actors in order to solve specific problems [35]. For constructivist authors, the institutional design of international agreements responds, rather, to a negotiation between the actors involved in a political game: the “what for” and the “for whom” [40,41,42].

To understand the potential impacts of COVID-19 on the institutional design of the 2030 Agenda we opt for the rational approach. Different reasons encourage this choice. First, the rational approach takes prominence in times of uncertainty about the state of the world, when the possibility of reforming international agreements is raised [35]. Second, when there are concerns about the behavior of the participants in agreements which might threaten the achievement of shared goals, reform based on rationalism is probable [43]. This is precisely the moment at which the 2030 Agenda finds itself after the recent transformation of the global scenario. Finally, the rational approach offers a suitable set of categories that can inform a useful systematization following the model proposed by Koremenos [36].

This framework [36] is based on a set of independent and dependent variables. The former refers to the problems, constraints on and characteristics of the main actors who play a role in the design of any international agreement. The latter are precisely the major dimensions of the institutional design of such agreements. In our case, as we have not performed a statistical analysis, instead of using the term “variable” we will speak of categories. On the one hand, contextual categories include what Koremenos calls independent variables and, on the other hand, design categories refer to Koremenos’ dependent variables. In some cases, these dimensions overlap, as is often the case with distribution and enforcement problems.

## 3. Results

### 3.1. Contextual Categories: How the World Is Being Shaped in the Post Covid-19 Era

In Table 2 we introduce the contextual categories [36]. These categories are classified in two broad sets: cooperation problems, and characteristics of states. Cooperation problems can be subdivided into interests and constraints.

#### 3.1.1. Cooperation Problems

Cooperation problems refer to a set of elements that can make it difficult for the actors in an agreement to cooperate. Some of these problems relate to the interests of those actors, and others relate to the underlying level of uncertainty, which acts as a constraint on cooperation. The analysis of cooperation problems is performed as follows: for each problem, the comments by authors who estimate that the problem will be more evident are introduced first, followed by arguments expressed against this assumption. Implications for the 2030 Agenda will be noted when necessary but are jointly and thoroughly addressed only in the discussion section.

Regarding distribution problems, the COVID-19 crisis has exposed those that underly any global agreement, such as that required to fight the virus and that of the 2030 Agenda. Some countries regard the situation as a win-lose game, where they will obtain more if others receive less (23). At the present moment, as is usually the case in any crisis of this nature, countries are fighting to gain a greater share of the available capacity, in terms of funds or medical supplies, for instance (24, 85). Some governments are banning or limiting exports (111), and even the EU is showing such behavior (107), despite the negative effects on all, especially the more fragile (80, 117).

However, some leaders and international organizations understand that undoubted distribution problem is surpassed by the global need to cooperate, initially through transmission, containment and mitigation policies (150) delivered worldwide, but in the long term, through effective development policies that reduce the vulnerability of our societies. Fortunately, there are examples of good practice that show the illusory nature of the distribution problem, in which scientists, international organizations, corporations, national governments and not-for-profit institutions around the world cooperate to find treatments and vaccines against the virus (120). If developed countries do not seek to protect the interests of the poorest, this type of menace (and those likely to come about in the future) will not be overcome because of the very nature of a pandemic (27). It will be made evident that there is a synergic potential when many countries agree on any kind of measure, such as economic stimuli (76, 96), and this will be more effective when no one defects. There are also proposals of financial formulae to provide support to developing countries that do not require funds from the developed world, such as the issuing of Special Drawing Rights by the International Monetary Fund (143).

Enforcement problems emerge when there is a prisoner’s dilemma situation, i.e., where a country is tempted to defect from an existing agreement, assuming that other countries will keep cooperating. The temptation to defect increases if the free rider foresees a better outcome of the distribution problem. During recent years, and especially during this crisis, many countries have increased their nationalistic Hobbesian stance, undermining or defecting from the international consensus, under the assumption that many other countries will remain faithful to it (66). These responses damage not only the current willingness to cooperate but also the trust in future cooperation needed to protect our planetary common resources (34, 42), and might even undermine our capabilities to fight the virus (30).

Nevertheless, we have seen that being global entails certain risks (42): high interdependence in terms of production (global value chains) and international human movements have influenced the rapid expansion of the pandemic, so it is more than probable that a new order will emerge in which solutions to this interdependence must be found (61, 108, 140), perhaps with a relevant role played by international cooperation rather than the implementation of protectionists measures (88). Many voices are being raised against this selfish behavior (22), calling for a more cooperative and holistic approach (29, 145), especially in terms of emphasizing the importance of multilateral platforms (30) and the need to provide greater support to developing countries (30, 34), because of the need to make sure that they are able to manage this crisis (27, 33, 34, 38).

Commitment problems appear when governments’ best plans for the future might not be acceptable when that future arrives, but also when there are internal divisions that endanger a country’s fulfilment of an agreement. Most of developed countries, even those in the EU, are traditionally committed to international cooperation (13, 111), but the US especially, whose lower-income population layers are currently suffering the consequences of having been abandoned by the system (25), are unveiling support measures for their citizens, with funds that they are likely to withdraw from other SDGs and from their international cooperation budgets (85, 98, 132, 147). If the economic slowdown persists, the 2030 Agenda implementation will be negatively impacted (27, 34, 140), even though all countries have agreed to support it in the long term. Governments are asking their citizens to endure severe constraints in the present, for the sake of positive impacts that will be felt in the future (29). On the other hand, internal dissensions within a country (for instance, between the states and the federal government in the US, (85)) or within a bloc (for example, the EU, (89, 93)) reduce the strength of current commitments, because any movement in the equilibrium among the different forces at stake could lead to a defection.

Coordination problems are frequently stated in the documents analyzed. The current COVID-19 crisis is a global problem, and as such it requires a global response (75, 106, 132). Thus, coordination problems appear, as only one free rider is needed to sabotage the chances of defeating the virus (34, 117). Additionally, different state preferences (which will be reviewed below) hinder a common international approach to this menace and to development in general (104, 120). These obstacles can be reduced when we face the real threat of a global collapse. COVID-19 shows that the incentives to cooperate are greater than those to compete or defect, because the coronavirus is our common enemy (66). The urgent need to find a shared solution reduces the distributional nature of the crisis and its underlying coordination problems (34). For example, the coronavirus is making explicit the need for a consensus between the US and China to fight this threat (120).

Despite the disjointed response deployed so far (19, 85, 96), there are areas where stronger coordination is needed and possible, such as knowledge sharing about cases, treatments and vaccine development (38, 120), although in the latter case some competition among firms and countries could be key to spur needed research (85), and there are some promising initiatives, especially among health institutions (23) and in the fiscal and financial areas (70, 76, 102). However, the nature of this pandemic requires a systemic international approach, and there are potential win-win strategies in different fields (70, 143). Some experts explicitly extend this need for joint action to other issues, such as climate change or poverty (104), which are obviously interrelated because they exacerbate and are exacerbated by COVID-19 (126). Multilateralism and a stronger global governance are regarded as necessary steps that should be undertaken to deal with global systemic risks (34).

Norm exportation distorts the capacity of the world to cooperate, because geopolitical movements generate noise in the international arena, preventing the trust needed to find global agreements. In the COVID-19 crisis this distortion is increasing because of the behavior of some major countries, such as China and Russia (44, 66, 68, 131, 150), who are using it as a way to increase their influence on the world and undermine confidence in those institutions intended to solve the crisis. Another world superpower, the US, has renounced its leading role (99, 106, 124), with President Trump’s administration’s disdain for international organizations (127), as his decision to freeze funds to the World Health Organization (WHO) shows (72, 85). The other major actor in the international show, the EU, is unable to stir up a common response (89, 93), despite some voices calling for more cohesion (106) and prominence (99, 105, 127).

The uncertainty about the state of the world is related to the knowledge these actors have about the consequences of their own and other actors’ actions [35], but in a broad sense can be understood as environment turbulence [44], in terms of our ability to comprehend what is happening now and, especially, what is likely to happen, due to the increasing number, novelty, complexity and speed of the changes introduced by this crisis. In that sense, it is clear that the world is now more uncertain at different levels, and in different domains and geographical areas, with developing countries being more likely to suffer the greatest impacts (13, 17, 19, 34, 53, 125). The major drivers of this uncertainty are related not only to the health domain but also to political (89), economic (38), social (108) and technological (92) categories, for example, with multiple interconnections between them (121).

Some experts claim that many of these trends were already latent, and the virus has merely intensified them (15, 141, 145). For example, the already existing financial market instability in some countries will increase (115), migrations will be exacerbated, despite their initial slowdown due to mobility restrictions (108), and our ability to fight climate change will be reduced (27, 29). Some terms used by experts, such as “chaos” (50) or “extremely disruptive” (99), reflect the feeling that perhaps we will never recover the world we used to know.

One source of uncertainty is the multiplier and systemic effect of the coronavirus (15, 150), affecting not only our health (especially in impoverished countries and households) but also, and especially, our economy (19, 134, 151), lifestyles (48) and social relations (2). Some experts label as unprecedented the collapse that COVID-19 can induce because it is provoking both a demand and a supply shock (152), with a future length (25, 152) and consequences difficult to foresee (125, 152). Another source of uncertainty is the interconnectedness of our world, which increases the speed, extension and depth of these impacts (22).

Some countries are showing disruptive conduct that hides their real intentions and behavior and helps to increase the level of uncertainty about the state of the world (7, 19), such as, but not only, China and Russia, which seem to be deploying their disinformation arsenal to further destabilize democratic regimes and extend their influence on the world (44, 61, 67, 68, 112, 124), thus eroding trust and hindering international organizations’ efforts (some of which have even been cyberattacked during the worst moments of the crisis, (87)) which are needed to deal globally with the coronavirus (61, 112). On the other hand, the consequences of COVID-19 will make it more difficult to properly manage current and future threats (105).

In this situation, there is great uncertainty about preferences, because it is more difficult to know what the other actors truly want to achieve, because of the disinformation already quoted, and also because countries, international organizations and regions are not monolithic institutions but show internal dissensions (89). The motivations of China, when they offer help to other countries, are suspicious, being tainted by their will to exert global leadership on the world, as said earlier. A specific source of uncertainty, given the relevance of the US as an international actor, hovers around President Trump, whose preferences and behavior (especially in an election year, (124)), and thereby the consequences of this, are unpredictable (85, 124).

Our confidence in essential institutions, such as science and international organizations, has been systematically undermined by discrediting campaigns orchestrated by irresponsible politicians and by conflicting points of view about the causes and solutions to the pandemic that different scientific bodies hold (4, 9). As a consequence, we are uncertain about the real motivations of our leaders and institutions (144).

#### 3.1.2. Characteristics of States

The characteristics of states are a set of categories related to the actors that participate in an agreement and that will be determinants of its institutional design. In the case of the 2030 Agenda, changes in different states caused by COVID-19 may require modifications to the original design to cope with the new situation. As with cooperation problems, changes and impacts in the characteristics of the states will be introduced in sequence. A holistic interpretation, compulsory in rational analyses, will be addressed in the following section.

A first conclusion elicited from the analyzed documents is that the coronavirus crisis is going to exacerbate power asymmetries already existing in the world, increasing economic inequalities among and within nations, eroding the international political legitimacy of major players in the global arena, the US, for instance, and causing a shift of power towards actors such as China (93, 106). 

Developing countries, and not necessarily those with more cases, will be the main losers here (16, 19, 34, 53, 65, 134, 141, 143, 148), mostly because of their structural weaknesses that expose them more to the crisis and prevent them from being able to deal with it, e.g., excessive debt, low external reserves, high poverty rates, high dependence on remittances, tourism and exports, under-resourced health and social care services already overwhelmed by other pandemics, such as AIDS or malaria (5, 8), high-density population settlements, political corruption and weak institutions (26, 63, 109, 150, 152). Some of the measures proposed to deal with coronavirus are not applicable in these countries, such as social distancing and lockdowns (16, 17). However, there will also be winners among the developing countries. The new economic order could imply a shift to more technology-intensive activities, including mobile tracking and other “tech” solutions, thus benefiting countries such as Singapore, Indonesia and Vietnam (115).

Regarding domestic regime-type asymmetries, experts fear a general drift towards more autocratic regimes, a trend that was evident before the outbreak of the pandemic (19, 66, 68, 69), and not only those states already showing certain levels of authoritarianism are likely to lose democratic freedoms (7, 131). The fearful populations of democratic countries will be willing to accept more political control and citizen surveillance if these guarantee a better fight against pandemics (50). However, it is not clear that authoritarian countries are better prepared to deal with COVID-19 (29, 59, 69).

Preference heterogeneity reflects the differences in actors’ interests. It is not new that countries and other stakeholders in the international arena differ on many substantial issues (51, 57, 59, 61, 104), and these differences are going to grow. There are clear geopolitical divisions that impede a global response to the crisis (120). In the EU, the fight between creditor and debtor countries is hindering a coordinated response (124). Nevertheless, there are also incentives to coordinate preferences (125), as has been said earlier.

A clear sign of preference heterogeneity is the responses of different countries to the crisis, according to their traditional way of understanding the international game. For example, some have suppressed information, others have used turmoil to tighten their power, some voices have called for more global governance, and there are those who forecast the return to unilateralism (22, 34, 70). Several governments have put public health before other concerns, but that has not always been the case (4, 71, 98). Support for international organizations is another field in which differences appear (72).

We can say that the main changes in the characteristics of states revolve around three main issues. First, although all countries are negatively affected by the pandemic, developing countries will suffer the worst, and the distribution of power in the world will reflect this impact. Second, authoritarianism could be seen by some states as a more effective regime to fight the virus, and thus democracy, at least in our liberal model, will lose supporters. Third, the already existing differences between countries in terms of preferences, especially those linked to health, will be increased.

In summary, cooperation among states is more difficult under the current circumstances, when distribution, enforcement and cooperation problems are increasingly evident, when some countries are trying to take advantage of the situation to extend their influence over the rest of the world and when there is more uncertainty. The pandemic will exacerbate existing differences and inequalities among and within states, with the danger of a generalized drift towards higher authoritarianism, all of which introduces noise to the system. However, there are also great incentives to coordinate actions, because otherwise the pandemic will not be defeated, and the most vulnerable will suffer.

Although experts do not explicitly link these changes in contextual categories to design categories or, in other words, to how the design of the 2030 Agenda should be modified to deal with them, they suggest ideas that could illustrate how this design could evolve in the context of uncertainty and conflicting interests among states, especially regarding the Global Health goals.

### 3.2. Design Categories: Institutional Design of the 2030 Agenda and the Global Health Goal

How will this new situation of delicate equilibrium affect the design of the 2030 Agenda, according to experts? How will the Global Health goal be pursued in this shifting scenario? To structure the analysis, the dimensions are organized as a set of design categories [36] and reflected in Table 3.

#### 3.2.1. Membership Rules

It is widely accepted that the 2030 Agenda requires a global partnership for its implementation, where not only governments, but also the private sector, civil society organizations, international organizations and other actors are summoned to participate [1]. Therefore, initially, there are different types of actor, government and institution, and the main distinction made regarding the former refers to the level of development of their society, with a special responsibility of the developed world towards the most deprived.

It seems that the prominence of governments will be reinforced as the main actors able to provide a viable solution to the problems posed by the pandemic (59, 80). However, the private sector can play a relevant role in the crisis (56, 59), both directly and through public-private partnerships (4, 87). It is also clear that the involvement of civil society groups, the scientific community and other types of international organizations is paramount (4, 7, 12, 56, 120). As a result, the membership rules of the 2030 Agenda should change to recognize different levels of participants with dissimilar rights and responsibilities. Experts are calling for a reform of the multilateral system to make it more effective, and some bestow on the G20 a special leading and coordinating role (96, 148), as will be seen below.

#### 3.2.2. Flexibility

Experts do not clearly address this topic, and no specific comments were found that could be classified under this dimension. Therefore, there are no clear proposals about whether the 2030 Agenda should be adjusted to include more flexibility clauses or perhaps a shorter time horizon in order to adapt to the main changes foreseen in the contextual categories.

#### 3.2.3. Centralization

In an increasingly interdependent world, experts agree that international institutionalized governance is the only way to manage the global crisis (17, 33, 34, 70) and to protect the health of the weak (23, 30, 72). The new governance arrangements proposed should make it possible to take collective action, pool sovereignty and centralize tasks to deal with problems such as this pandemic, so the delegation of monitoring, information and coordination to a single focal entity is needed for more effective action, at national and international levels (12, 17, 56, 75). The 2030 Agenda is regarded as a unique opportunity for stepping up international cooperation (30). Unfortunately, geopolitical divisions impede anything like a world government. Even the EU is unable to deploy a coordinated response (89, 93, 106) to COVID-19, despite the qualified voices requesting it (89, regarding French premier Macron).

The WHO and other international institutions have not been able to offer a well-managed response to the pandemic and should be reformed to wield greater centralized powers that allow for a better response to this type of threat (72, 124, 148).

Many experts propose the G20 as an important centralizing agent (74, 75, 117, 148), despite its scarce accomplishments so far (34, 96). Therefore, in general, there is an agreement about greater centralization in multilateral institutions to deal more effectively with the menace of this and future pandemics.

Following the spirit of the Agenda, however, some experts also underscore the importance of decentralization to deal with the pandemic. Community-based networks, local government empowerment and other forms of people participation are needed for an effective response to the virus (8, 9, 59, 71, 138, 142). Therefore, a balance between these two forces seems to be key here, with decentralized structures at local level and centralized institutions at international level.

#### 3.2.4. Scope

Will the scope of issues included in the 2030 Agenda be affected by the pandemic and, even more relevant, should they be? What about global health issues? Experts mostly agree that, given the systemic nature of health, holistic approaches to deal with the coronavirus should be encouraged (7, 150). Many voices warn about the links between poverty and health (16) in a diverse array of areas. For example, health systems in poor countries are under-resourced, so the impact of the disease is expected to be greater (5, 17, 150). Quarantines, lockdowns, and other social distancing measures are more difficult to implement in developing countries, where thousands of people gather in crowded slums and refugee camps with limited space and scarce sanitation services (5, 28). The outcomes of these constraints are more severe for these countries due to their lack of safety nets and other types of support (150, 60). Poor countries show a higher dependence on sectors that are particularly vulnerable to these impacts, such as tourism or manufacturing (88, 140). Malnourishment weakens the immune system and increases the risk of morbidity and mortality (150). Corruption, casual behavior, lack of transparency and bureaucracy are burdensome obstacles to fighting the virus in these countries (31, 45, 77).

There is also a close link between health, poverty and the environment (5, 11), as poor people are more likely to live in polluted environments that weaken their health and make them more susceptible to infection. Women and children are also more vulnerable to the effects of the crisis, so any arrangement intended to deal with the pandemic should include special measures to protect them (133, 129, 150).

Earlier paragraphs show the systemic nature of this shock, and for some experts it is clear that a multiple-issue approach, and not only health-oriented, should be followed if we are to succeed in our fight against this and future pandemics (32, 72). COVID-19 affects all SDGs (30), and if the world had advanced more, we would be better prepared to mitigate its impacts (61). This argument could be used as a wake-up call to challenge the status quo and design a new international order able to deal more effectively with pandemics (14).

Unquestionably, the fight against the coronavirus is going to detract funds from other causes, and not only related to other health issues (16, 23, 93). Table 4 shows which targets under Goal 3 are related to COVID-19, so we may think that the rest are likely to receive less attention in the future. The resources allocated to deal with the pandemic and its effects will slow down the implementation of the 2030 Agenda (140).

At national and international levels, health-related goals could be prioritized over other social issues, which might seem less urgent (30, 80), especially in the case of developing countries (98). The forecast increase in the relative and absolute number of poor people (33) will also make it more difficult to achieve the proposed goals. 

#### 3.2.5. Control

As with flexibility, control is not addressed by experts. Again, nothing is said about whether the 2030 Agenda should include control clauses to incorporate differences among states, but it is not inconceivable to think of some countries demanding more control over the decision-making processes related to the Agenda if they are going to be asked to contribute more funds.

## 4. Discussion

In our analysis, we have selected and reviewed a sample of documents that reflect the ideas of a relevant group of experts about the impact of COVID-19 on the framework provided by the 2030 Agenda to promote the governance of Global Health issues. Our results show how there are constraints in terms of uncertainty that condition and threaten the necessary cooperation between countries and, subsequently, the role of the 2030 Agenda in addressing current and future development challenges, specifically the Global Health goal of the SDGs. There are conflicting opinions about the key categories, but there are some points of agreement. 

One first overall result points out how the return to a nationalistic defense of each country’s health system and citizens is not a solution, despite the initial reaction of certain world leaders. This type of crisis requires a joint action that overcomes the selfish interests of the traditional state-nation and is able to see beyond, perhaps reformulating multilateralism to adapt to this new reality. A return to nationalistic approaches could also undermine the capabilities of developing countries in ensuring public health coverage.

There is a high level of uncertainty about how the world will change. There is the risk of a slide towards more autocratic regimes and the loss of basic human rights in order to wield more effective weapons against the virus. Some autocratic countries are using the turmoil of these initial moments to export their model and undermine multilateralism. If our model of international cooperation and liberal democracy is to prevail, democratic countries and international organizations have to show that there are ways to defeat the coronavirus without eroding those basic rights.

Experts find divergence between what some governments are doing and what, in their view, should be done. The fact that the answers to the crisis remain under the monopoly of governments not only shows the prevalence of national interests, but also questions the principles of the social construction of multilateralism. However, this finding would need to be further explored in future research, given its potential impact on the future multilateral agenda, as it illustrates a major threat to global governance.

One limitation that must be taken into consideration refers to the selected period of analysis. In our study we wanted to highlight the short-term reactions to the pandemic, immediately after it was declared as such by the World Health Organization. The reason for this is that we wanted to point out the weaknesses of the 2030 Agenda and its exposure to unexpected shocks to the international equilibrium, which in the future can come in the form of new diseases or environmental impacts due to climate change. This focus might have slightly biased the representativeness of the voices from developing countries, as the pandemic reached these only later on time. This is a limitation to the study, and also a potential future line of research.

Despite this, it seems clear that we find ourselves in a very different situation from that which served as the setting for the design of the 2030 Agenda. The results of our analysis show how these changes could have a significant impact on the main design elements of the Agenda, which leads us to believe that its implementation could be more contingent. Should the 2030 Agenda be reformulated? In what terms? Although experts have hinted at some suggestions, a more reflective proposal is needed. In our opinion, the rational model we have used here provides a clear structure for analysis in such future research.

## 5. Conclusions

It is worth noting that the scenario presented in this study could dramatically change if an effective vaccine or treatment is found in the short term. However, let us not deceive ourselves; the post-COVID-19 world is not going to be the same. Our weaknesses, as well as our strengths, have been exposed, and our multilateral system clearly needs to be adapted to this new reality.

For instance, the rational approach to institutional design could be used to propose how the 2030 Agenda should be changed and in what direction. As the literature highlights, the design of international agreements is the result of rational and deliberate interactions between states and other international actors in order to solve specific problems. Following these authors, design of such agreements must be aligned with the problems it seeks to resolve. Therefore, the evidence gathered by our analysis can inform the debate on how the 2030 Agenda could respond effectively to the complex problems brought about by the COVID-19 pandemic, in order to fulfill the expectations of the states that participate in it.

There are many factors that might influence the results of an international agreement such as the 2030 Agenda. Obviously, adapting the design of the Agenda to the challenges posed by the recent evolution of the international arena is not enough. However, if this reform is not undertaken, most likely the world will not attain the SDGs such as Global Health, because we will be looking to the future with eyes of the past.

## Figures and Tables

**Table 1:** Top 15 Think tanks in the category International Development Policy.

Rank	Think Tank	Nationality
Top	Korea Development Institute	South Korea
1	Institute of Development Studies	United Kingdom
2	Brookings Institution	United States
3	German Development Institute	Germany
4	Wilson Center	United States
5	Chatham House	United Kingdom
6	Asian Development Bank Institute	Japan
7	Center for Strategic and International Studies	United States
8	Danish Institute for International Studies	Denmark
9	Council on Foreign Relations	United States
10	Fundação Getulio Vargas	Brazil
11	Center for International Development	United States
12	Development Research Center of the State Council	China
13	Friedrich Ebert Foundation	Germany
14	World Institute for Development Economics Research	Finland
15	Overseas Development Institute	United Kingdom

Source: 2019 Global go To Think Tank Index [18].

**Table 2:** Classification of contextual categories and their prevalence in documents.

Contextual Categories (Number/% of Documents Addressing the Topic)		
Cooperation problems (84/70.6%)	Interests (63/52.9%)	Distribution problems (19/16.0%)
		Enforcement problems (34/28.6%)
		Commitment problems (21/17.6%)
		Coordination problems (31/26.1%)
		Norm exportation (17/14.3%)
	Constraints (58/48.7%)	Uncertainty about the state of the World (43/36.1%)
		Uncertainty about behavior (20/16.8%)
		Uncertainty about preferences (13/10.9%)
Characteristics of States (58/48.7%)		Number (0/0.0%)
		Power asymmetries (33/27.7%)
		Domestic regime-type asymmetries (24/20.2%)
		Preference heterogeneity (26/21.8%)

Source: Author’s own elaboration based on Koremenos [36].

**Table 3:** Prevalence of design categories in documents.

Design Categories	Number and % of Documents
Membership rules	10/8.4%
Flexibility	1/0.8%
Centralization	32/26.9%
Scope	48/40.3%
Control	0/0.0%

Source: Author’s own elaboration based on Koremenos [36].

**Table 4:** Analysis of Sustainable Development Goal (SDG) 3 according to its relation to COVID-19.

Goal 3 Targets Related to COVID-19	Goal 3 Targets NOT Related to COVID-19
Infectious diseases (target 3.3)	Reproductive, maternal, newborn and child health (targets 3.7, 3.1, 3.2)
Health systems and funding (targets 3.8, 3.B, 3C, 3D)	Non-communicable diseases, mental health and environmental risks (targets 3.4, 3.5, 3.6, 3.9)
	Health systems and funding (target 3.A)

Source: Author’s own elaboration based on the 2030 Agenda [1].

## Appendix A

**Table A1:** Documents used for the analysis after filtering.

Code	Date	Title	Author’s Name	Source
1	16 March 2020	Countering the Coronavirus: can people remain safe and still practice their faith?	Mariz Tadros	Institute of Development Studies
2	15 April 2020	Covid-19—the experience of living a pandemic rather than researching one	Annie Wilkinson	Institute of Development Studies
3	8 April 2020	Covid-19 in low-income countries—we need rapid learning about effective handwashing initiatives	Jamie Myers	Institute of Development Studies
4	3 April 2020	COVID-19 may be the ultimate test of science and policy partnerships	James Georgalakis	Institute of Development Studies
5	6 April 2020	Covid-19 reveals and further increases inequalities in water and sanitation	Lyla Metha et al.	Institute of Development Studies
6	2 April 2020	Excessive health damage from Covid-19 will be followed by excessive wealth damage unless governments act now	Michael Lipton	Institute of Development Studies
7	8 April 2020	Fear of a fragile planet	Naomi Hossain	Institute of Development Studies
8	25 March 2020	Lessons from Brazil for the global response to COVID-19	Alex Shankland	Institute of Development Studies
9	16 March 2020	Science, uncertainty and the COVID-19 response	Ian Scoones	Institute of Development Studies
10	24 March 2020	Strengthening Brazilian partnerships in the face of Covid-19	Alex Shankland and Rachel Dixon	Institute of Development Studies
11	21 March 2020	With climate change impacts accelerating, we need to re-think the human right to water	Lila Metha, Claudia Ringler and Shiney Varghese	Institute of Development Studies
12	1 April 2020	Lessons from Covid-19: building more effective health services for a complex future	Gerald Bloom	Institute of Development Studies
13	20 March 2020	COVID-19—a social phenomenon requiring diverse expertise	Haylee MacGregor et al.	Institute of Development Studies
14	23 March 2020	Precarious and informal work exacerbates spread of coronavirus	Ayako Ebata et al.	Institute of Development Studies
15	14 April 2020	How COVID-19 will change the nation’s long-term economic trends, according to Brookings Metro scholars	Mark Muro et al.	Brookings Institution
16	23 March 2020	A mortality perspective on COVID-19. Time, location, and age	Katharina Fenz and Homi Kharas	Brookings Institution
17	11 April 2020	Africa in the news. COVID-19 impacts African economies and daily lives; clashes in the Sahel	Dhruv Gandhi, Anna Schaeffer, and Payce Madden	Brookings Institution
18	4 April 2020	Africa in the news. Impacts of COVID-19 on African economies and elections updates	Christina Golubski and Anna Schaeffer	Brookings Institution
19	26 March 2020	Brookings experts on the implications of COVID-19 for the Middle East and North Africa	Tarik M. Yousef, Ranj Alaaldin, Geneive Abdo et al.	Brookings Institution
20	27 March 2020	COVID-19. Does India have enough Doctors? An analysis of growing COVID-19 patients and existing medical capacity	Prachi Singh, Dweepobotee Brahma, and Sikim Chakraborty	Brookings Institution
21	24 March 2020	COVID-19. Is India’s health infrastructure equipped to handle an epidemic	Prachi Singh, Shamika Ravi, and Sikim Chakraborty	Brookings Institution
22	12 March 2020	COVID-19 is a reminder that interconnectivity is unavoidable	Morgan D. Bazilian and Samantha Gross	Brookings Institution
23	2 April 2020	Ebola lessons for fighting COVID-19	Ngozi Okonjo-Iweala	Brookings Institution
24	8 April 2020	Understanding the impact of the COVID-19 outbreak on the Nigerian economy	Chukwuka Onyekwena and Mma Amara Ekeruche	Brookings Institution
25	3 April 2020	Who are the workers already impacted by the COVID-19 recession	Alan Berube and Nicole Bateman	Brookings Institution
26	6 April 2020	How the EU and rising powers can shape their future sustainably	Sven Grimm et al.	German Development Institute
27	2 April 2020	Curb your enthusiasm: Corona may slow down multilateral process, but must not derail global climate policy	Clara Brandi et al.	German Development Institute
28	9 April 2020	How the corona crisis is calling into question the right to the city	Eva Dick	German Development Institute
29	1 April 2020	Parallels between the corona pandemic and climate change	Hanna Fuhrmann and Sascha Kuhn	German Development Institute
30	3 March 2020	Coronavirus as an opportunity for international cooperation	Paul Marschall and Wulf Reiners	German Development Institute
31	2 April 2020	What we can learn from and about Africa in the corona crisis	Michael Roll	German Development Institute
32	30 March 2020	How we will need to tackle climate migration post-coronavirus	Benjamin Schraven	German Development Institute
33	26 March 2020	Why social protection is crucial in the corona crisis	Christoph Strupat, Francesco Burchi and Daniele Malerba	German Development Institute
34	1 April 2020	Lessons for Global Cooperation from the COVID-19 Pandemic	Gianluca Grimalda	German Development Institute
35	3 April 2020	An Uncertain Recovery	Verónica Ortíz-Ortega	Wilson Center
36	19 March 2020	Canada’s Response to Coronavirus	Mariana Sánchez-Ramírez	Wilson Center
37	16 April 2020	COVID-19 and the Threat to the North American Economy	James Haley	Wilson Center
38	20 March 2020	COVID-19: The Global Evil	Verónica Ortíz-Ortega	Wilson Center
39	17 March 2020	Exploring the Complexity of Pandemics Through Play	Elizabeth Newbury	Wilson Center
40	23 March 2020	Home-Clinic to Face COVID-19 in Mexico	Luis de la Calle	Wilson Center
41	14 April 2020	How Will Southeast Asia’s Conflict Zones Fare in 2020 and beyond?	Prashanth Parameswaran	Wilson Center
42	23 March 2020	If Games are Postponed, Japan Can Still Bring a World in Pain Together	Shihoko Goto	Wilson Center
43	30 March 2020	Mexico’s Energy Policy in times of Covid-19?	Lourdes Melgar	Wilson Center
44	1 April 2020	Moscow-Driven “Forced Reintegration” Scenario Endangers Ukraine’s National Security	Igor Popov	Wilson Center
45	17 March 2020	News Roundup: The MENA Region in the Time of COVID-19	Merissa Khurma and Alexander Farley	Wilson Center
46	24 March 2020	Projected Impact of COVID-19 on Ukraine’s Economy	Adrian Prokip	Wilson Center
47	8 April 2020	Rebuilding Public Trust in International Aviation: An Opportunity for U.S.-Canada Leadership	Solomon Wong and Marcelo Garcia	Wilson Center
48	26 March 2020	Reports from North America’s Borders: Experts React to New COVID-19 Travel Restrictions	Jon Barela et al.	Wilson Center
49	17 March 2020	Russia and Eurasia Respond to the Pandemic	Morgan Jacobs	Wilson Center
50	7 April 2020	Russia’s “Special Path” in the Global Pandemic	Sergey Parkhomenko	Wilson Center
51	27 March 2020	Russia’s Chinese Dream in the Era of COVID-19	Emily Couch	Wilson Center
52	10 April 2020	South Korea’s Parliamentary Elections: Key Variables and Their Implications	Soojin Park	Wilson Center
53	27 March 2020	Survival: Venezuela and the Coronavirus	Beatriz García-Nice	Wilson Center
54	23 March 2020	The First Days of COVID-19 in Ukraine	Yuriy Vakhel	Wilson Center
55	9 April 2020	Ukraine Quarterly Digest: January-March 2020	Andrian Prokip	Wilson Center
56	7 April 2020	Using tech to fight the virus: How much privacy are South Koreans relinquishing in the battle against COVID-19?	Jean H. Lee	Wilson Center
57	2 April 2020	Washington versus Moscow: Official responses to Covid-19	Grigory Vaipan	Wilson Center
58	24 March 2020	What Lies Behind Russia’s Coronavirus Containment Effort	Judy L. Twigg	Wilson Center
59	31 March 2020	What the U.S. Can Learn from Asia’s Coronavirus Response	Alex Long	Wilson Center
60	24 March 2020	Will COVID Redefine the East Asian Miracle?	Shihoko Goto	Wilson Center
61	24 March 2020	Wilson Center Experts Weigh in on the Coronavirus	Cynthia J. Arnson, Diana Villiers, Christopher Sands	Wilson Center
62	10 April 2020	Women’s Choice: COVID-19 or an Abusive Partner	Olimpiada Usanova	Wilson Center
63	17 March 2020	What Coronavirus Means for South Asia	Michael Kugelman	Wilson Center
64	27 March 2020	Ukrainians Keep Their Composure During the COVID-19 Epidemic	Semen Gluzman	Wilson Center
65	10 April 2020	Downtrodden in a Shut Down	Aníbal Nicolás Saldías	Wilson Center
66	9 April 2020	COVID-19 Brings Human Rights into Focus	Sonya Sceats	Chatham House
67	29 March 2020	In a COVID-19 World, Russia Sticks to International Distancing	Mathieu Boulègue	Chatham House
68	9 April 2020	Beware Russian and Chinese Positioning for After the Pandemic	Keir Giles	Chatham House
69	31 March 2020	Coronavirus and the Future of Democracy in Europe	Hans Kundnani	Chatham House
70	16 April 2020	How to survive the pandemic	Creon Butler	Chatham House
71	16 March 2020	America’s Coronavirus Response Is Shaped By Its Federal Structure	Leslie Vinjamuri	Chatham House
72	15 April 2020	Blaming China Is a Dangerous Distraction	Jim O´Neill	Chatham House
73	6 April 2020	Can Morocco Effectively Handle the COVID-19 Crisis?	Mohammed Masbah and Anna Jacob	Chatham House
74	16 March 2020	Coronavirus: All Citizens Need an Income Support	Jim O´Neill	Chatham House
75	15 March 2020	Coronavirus: Global Response Urgently Needed	Jim O´Neill, Robin Niblett, Creon Butler	Chatham House
76	18 March 2020	Coronavirus: Why The EU Needs to Unleash The ECB	Pepijn Berssen	Chatham House
77	7 April 2020	COVID 19: Assessing Vulnerabilities and Impacts on Iraq	Renad Mansour, Mac Skelton and Abdulameer Mohsin Hussein	Chatham House
78	15 April 2020	Emerging Infections in Perspective: Novel Coronavirus and H7N9 Influenza	David Heymann	Chatham House
79	2 April 2020	Emerging Lessons From COVID-19	Jim O´Neill	Chatham House
80	26 March 2020	Let’s Emerge From COVID-19 with Stronger Health Systems	Robert Yates	Chatham House
81	24 March 2020	The G20’s Pandemic Moment	Jim O´Neill	Chatham House
82	16 April 2020	Belarusians Left Facing COVID-19 Alone	Ryhor Astapenia and Anais Marin	Chatham House
83	8 April 2020	COVID-19 and the Iranian Shadows of War	Sanam Vakil	Chatham House
84	31 March 2020	COVID-19 Impact on Refugees is Also Political	Lina Khatib	Chatham House
85	6 April 2020	In Search of the American State	Leslie Vinjamuri	Chatham House
86	1 April 2020	Predictions and Policymaking: Complex Modelling Beyond COVID-19	Yasmin Afina and Calum Inverarity	Chatham House
87	2 April 2020	Supporting NHS Cybersecurity During COVID-19 is Vital	Joyce Hakmeh	Chatham House
88	15 April 2020	Why an Inclusive Circular Economy is Needed to Prepare for Future Global Crises	Patrick Schröder	Chatham House
89	6 April 2020	An Eroding European Union	Heather A. Conley	Center for Strategic and International Studies
90	15 April 2020	Australia Goes Hard and Goes Early on Covid-19	Parick Gerard Buchan	Center for Strategic and International Studies
91	15 April 2020	China’s External Sector: Imagining the Post- Covid-19 Reality	Kevin Nealer	Center for Strategic and International Studies
92	13 April 2020	China’s Digital Silk Road after the Coronavirus	Jude Blanchette and Jonathan E. Hillman	Center for Strategic and International Studies
93	27 March 2020	Competition or Coordination: Coronavirus in the Developing World	Daniel F. Runde; Sundar Ramanujan	Center for Strategic and International Studies
94	3 April 2020	Cooperation, Not Fear, Keeps the Food Supply Chain Secure	Caitlin Welsh	Center for Strategic and International Studies
95	18 March 2020	Coronation, Coronavirus, and the Economy: The Economic Backdrop of a Fifth Putin Term	Cyrus Newlin	Center for Strategic and International Studies
96	3 April 2020	Covid-19 and Value Chains: Diminishing Returns from Trade Policy	Scott Miller	Center for Strategic and International Studies
97	10 April 2020	Covid-19 at Sea: Impacts on the Blue Economy, Ocean Health, and Ocean Securit	Whitley Saumweber and Amy K. Lehr	Center for Strategic and International Studies
98	6 April 2020	Covid-19 Exposes Latin America’s Inequality	Linnea Sandin	Center for Strategic and International Studies
99	18 March 2020	COVID-19 Is an African Political Crisis as Much as a Health and Economic Emergency	Judd Devermont	Center for Strategic and International Studies
100	25 March 2020	COVID-19 Is Attacking Our Defense Supply Chains and Our Nation’s Security	Andrew Philip Hunter	Center for Strategic and International Studies
101	6 April 2020	Donald Trump Is Right. We Need “BIG & BOLD” Infrastructure Spendin	Lachlan Carey	Center for Strategic and International Studies
102	2 April 2020	Emergency Planning for OPEC States	Ben Cahill	Center for Strategic and International Studies
103	26 March 2020	Empowering Women through Skills and Workforce Development	Daniel F. Runde; William J. Garvelink and Janina Staghun	Center for Strategic and International Studies
104	9 April 2020	Energy and Emissions after Covid-19: A First Cut	Sarah Ladislaw and Nikos Tsafos	Center for Strategic and International Studies
105	13 April 2020	Europe Is at War with the Coronavirus. Where Does That Leave European Defense?	Quentin Lopinot	Center for Strategic and International Studies
106	23 March 2020	Europe’s Coronavirus Test	Quentin Lopinot and Donatienne Ruy	Center for Strategic and International Studies
107	16 April 2020	Find My Friends in a Pandemic: the Future of Contact Tracing in America	Anna Carroll	Center for Strategic and International Studies
108	25 March 2020	Five Ways COVID-19 Is Changing Global Migration	Erol Yaiboke	Center for Strategic and International Studies
109	13 April 2020	Latin America: On the Verge of an Unprecedented Turn in the Covid-19 Pandemic?	Michael A. Matera	Center for Strategic and International Studies
110	2 April 2020	NATO Responds to the Covid-19 Pandemic	Rachel Ellehuus	Center for Strategic and International Studies
111	2 April 2020	Pandemic Pandemonium: How the Virus Could Change the Trading System	William Alan Reisch	Center for Strategic and International Studies
112	31 March 2020	Putin and the COVID Crisis: Instability as Opportunity	Iain King and Rachel Ellehuus	Center for Strategic and International Studies
113	2 April 2020	Seeking a Path to Europe, Refugees and Migrants Ultimately Turned Back by Covid-1	Erol Yaboke and Joseph S. Bermúdez Jr.	Center for Strategic and International Studies
114	9 April 2020	Supporting Mozambique’s Response to the Growing Insurgent Threat in Cabo Delgad	Emila Columbo	Center for Strategic and International Studies
115	14 April 2020	The Economic Toll of Covid 19	Amy Searight	Center for Strategic and International Studies
116	30 March 2020	The End of OPEC or a New Beginning?	Sara Ladislaw	Center for Strategic and International Studies
117	8 April 2020	The G20 Agreement the World Needs Now	Sara Ladislaw	Center for Strategic and International Studies
118	8 April 2020	The Mexican Government’s Response to Covid-19 Is Insufficient	Gladis MacKormick	Center for Strategic and International Studies
119	19 March 2020	Time to Call Off the Oil War	Frank A. Verrastro; Larry Goldstein and Albert Hermig	Center for Strategic and International Studies
120	20 March 2020	U.S.-China Relations and COVID-19: What Can Be Done Now	John L. Holden	Center for Strategic and International Studies
121	1 April 2020	Which Covid-19 Future Will We Choose?	J. Stephen Morrison and Anna Carroll	Center for Strategic and International Studies
122	7 April 2020	Will Covid-19 End the Age of Mass Protests?	Samuel Brannen	Center for Strategic and International Studies
123	8 April 2020	Fuel Shortages during Covid-19 in Venezuela	Moisés Rendón and Margarita Seminario	Center for Strategic and International Studies
124	15 April 2020	What’s on the Horizon for Covid-19	J. Stephen Morrison et al.	Center for Strategic and International Studies
125	31 March 2020	Geopolitical Scenarios for Asia after COVID-19	Michael J. Green	Center for Strategic and International Studies
126	27 March 2020	Africa and the Third Wave of Covid-19	Neil Anthony Webster	Danish Institute for International Studies
127	31 March 2020	Asia beyond China	Luke Patey	Danish Institute for International Studies
128	1 April 2020	COVID-19: A Looming humanitarian disaster for Somali East Africa	Abdirahman Edle Ali et al.	Danish Institute for International Studies
129	10 April 2020	Women This Week: The Gendered Effects of COVID-19	Maleeha Coleman et al.	Council on Foreign Relations
130	6 April 2020	At War With a Virus	Richard N. Haas	Council on Foreign Relations
131	16 March 2020	China and Coronavirus: From Home-Made Disaster to Global Mega-Opportunity	Joshua Kurlantzick	Council on Foreign Relations
132	7 April 2020	U.S. Coronavirus Response: Who’s In Charge of What?	Lindsay Maizland	Council on Foreign Relations
133	7 April 2020	COVID-19 and lockdowns Are women more affected?	Bina Agarwal	United Nations University (UNU-WIDER)
134	2 April 2020	Estimates of the impact of COVID-19 on global poverty	Andy Sumner et al.	United Nations University (UNU-WIDER)
135	10 April 2020	Age composition of population and Covid-19	Kunal Sen	United Nations University (UNU-WIDER)
136	8 April 2020	Is Mozambique prepared for a lockdown during the COVID-19 pandemic?	Sam Jones, Eva Maria Egger and Ricardo Santos	United Nations University (UNU-WIDER)
137	6 April 2020	To die from hunger or the virus An all too real dilemma for the poor in India	Marty Chen	United Nations University (UNU-WIDER)
138	16 March 2020	When COVID-19 comes to Africa	Arkebe Oqubay	United Nations University (UNU-WIDER)
139	14 April 2020	Countries facing Covid-19 debt need flexible financing: lessons from China	Arkebe Oqubay	United Nations University (UNU-WIDER)
140	1 April 2020	Covid-19 and trade: challenges ahead for Least Developed Countries and Small Island Developing State	Jodie Keane	Overseas Development Institute
141	1 April 2020	Covid-19: ‘we won’t get back to normal because normal was the problem’	Sara Pantuliano	Overseas Development Institute
142	20 March 2020	Covid-19: five lessons from Ebola	Sorcha O’Callaghan	Overseas Development Institute
143	18 March 2020	Financing for developing countries facing a coronavirus-sparked economic crisis	Jesse Griffiths	Overseas Development Institute
144	17 March 2020	Governments must catch up to curb the coronavirus pandemic	Arkebe Oqubay	Overseas Development Institute
145	26 March 2020	How coronavirus is accelerating a new approach to international cooperation	Annalisa Prizzon	Overseas Development Institute
146	9 April 2020	“Libya and pandemic politics in armed conflicts	Sherine El Taraboulsi-McCarthy	Overseas Development Institute
147	16 April 2020	Migrant key workers: Time to act	Marta Foresti	Overseas Development Institute
148	27 March 2020	The G20’s coronavirus action plan must help the poorest countries	Dirk Willem te Velde	Overseas Development Institute
149	7 April 2020	What research from conflict-affected countries can tell us about responses to Covid-19	Mareike Shomerus	Overseas Development Institute
150	2 April 2020	Hotspots of vulnerability in times of crisis	Vidya Diwakar	Overseas Development Institute
151	2 April 2020	How to scale up multilateral financing to face the Covid-19 crisis	Chris Humphrey	Overseas Development Institute
152	3 April 2020	The coronavirus pandemic and the governance of global value chains: emerging evidence	Jodie Keane	Overseas Development Institute

Source: Author’s own elaboration.
